# *JAG1* is correlated to suppressive immune microenvironment and predicts immunotherapy resistance in lung adenocarcinoma

**DOI:** 10.3389/fonc.2023.1091488

**Published:** 2023-02-27

**Authors:** Jing He, Lu Li, Lulu Lv, Xiaoyan Chen, Minghui Ge, Yong Ren, Xinyu Tang, Ping Liu, Wen Gao

**Affiliations:** ^1^ Department of Oncology, Jiangsu Province Hospital and Nanjing Medical University First Affiliated Hospital, Nanjing, China; ^2^ State Key Laboratory of Translational Medicine and Innovative Drug Development, Jiangsu Sincere Diagnostics Co., Ltd., Nanjing, China; ^3^ Nanjing Sincere Medical Laboratory Science Co., Ltd., Nanjing, China; ^4^ Department of Radiation Oncology, Jiangsu Province Hospital and Nanjing Medical University First Affiliated Hospital, Nanjing, China

**Keywords:** lung adenocarcinoma, angiogenesis, tumor immune microenvironment, glycolysis, JAG1

## Abstract

**Background:**

The current exploration of the tumor immune microenvironment is enthusiastic, but few studies explored the impact of angiogenesis on the immune microenvironment. Immunotherapy combined with anti-angiogenesis therapy has become one of the first-line treatment for lung adenocarcinoma. Our study aimed to explore the reasons for resistance of immunotherapy, and explore markers for immunotherapy combined with anti-angiogenesis therapy.

**Methods:**

First, by unsupervised clustering of 36 angiogenesis-related genes in lung adenocarcinoma patients from TCGA database, AGS1 and AGS2 groups were distinguished with significantly different clinical outcomes. Secondly, the immune microenvironment and metabolic characteristics were analyzed. Next, we used the GDSC and GEO database to analyze therapeutic responses. Then, through multivariate Cox regression, the hub gene: *JAG1*, significantly related to prognosis was selected, and further verified by multi-omics data. Finally, we validated that patient with high *JAG1* expression had a low immune-infiltrating tumor microenvironment through single-cell transcriptomic data.

**Results:**

Compared with the AGS1 group, AGS2 showed an immune “cold” phenotype with lower lymphocyte infiltration, and was associated with worse prognoses. At the same time, the immunosuppressive TGF-β response was significantly higher in AGS2. Furthermore, the glycolysis ability of the AGS2 was stronger than AGS1. The expression of *JAG1* was significantly higher in the AGS2, and was significantly negatively correlated with the degree of immune infiltration, accompanying with higher glycolytic capacity. The above results indicate that patients with high expression of *JAG1* may lead to immunosuppressive phenotype due to its strong glycolytic capacity, thus making immunotherapy resistance.

**Conclusion:**

Patients with high expression of *JAG1* enhanced glycolytic capacity was likely to cause suppressed immune microenvironment. *JAG1* may be a marker for resistance of immunotherapy. Combining anti-angiogenesis therapy could be considered to improve the prognosis of those patients.

## Introduction

Angiogenesis is the growth of blood vessels from the existing vascular bed. It is a constant process throughout life in both health and disease. In solid tumors, the complex biological process of angiogenesis is involved in sustaining the tumor microenvironment, growth, and metastatic dissemination (1).

Inhibition of vascular endothelial growth factor (VEGF) or VEGF receptors (VEGFR) is a common therapeutic strategy in oncology because VEGF is the critical mediator of angiogenesis in cancer. The first VEGF signaling pathway inhibitor was approved in 2004 (Bevacizumab) and has been approved for use in diverse solid tumors. VEGF inhibitors are still continuously investigated nowadays (2).

Bevacizumab was approved for the first-line treatment of patients with advanced non-squamous NSCLC in combination with chemotherapy in 2006 based on the results of phase III trials ECOG4599, which demonstrated bevacizumab added to chemotherapy improved both progression-free survival (PFS) and overall survival (OS) versus chemotherapy alone (3).

It is well-known immune checkpoint inhibitors (ICIs) which block coinhibitory molecules such as CTLA-4, programmed cell death protein-1 (PD-1), and the related programmed death-ligand 1 (PD-L1) have changed the treatment scenario of advanced NSCLC first-line setting as breakthroughs. Both the U.S. Food and Drug Administration and the China National Medical Products Administration (NMPA) approved over ten indications of ICIs each for NSCLC clinical practice, including PD-1inhibitor (pembrolizumab/nivolumab/camrelizumab/tislelizumab/sintilimab), PD-L1 inhibitor (atezolizumab/durvalumab/sugemalimab), CTLA-4 inhibitor (ipilimumab). Most of which are immune combination therapy (4–6).

Although immunotherapy has proven to be an effective and important new strategy for the management of LUAD patients, only a part of patients benefits from immunotherapy (7). This phenomenon may be attributed to the varied heterogeneity of the immune microenvironment among individuals (8). Therefore, it is important to further explore the regulatory mechanisms of the tumor immune microenvironment to optimize the management of immunotherapy.

Phase 3 randomized trial IMpower150 compared first-line therapy with bevacizumab plus carboplatin plus paclitaxel (BCP) versus atezolizumab plus BCP (ABCP) for patients with metastatic non-squamous NSCLC. Median OS was 4.5 months longer in the ABCP arm versus in BCP arm (19.2 months *vs* 14.7 months; hazard ratio for death, 0.78; 95% CI, 0.64 to 0.96; *P*=0.02). PFS was longer in the ABCP arm versus in BCP arm (8.3 *vs*. 6.8 months; HR,0.62; 95% CI, 0.52–0.74; *P* <.001). The result demonstrated that PD-L1 inhibitor (atezolizumab) added to VEGF inhibitor (bevacizumab) and chemotherapy significantly improved PFS and OS among metastatic non-squamous NSCLC compared to VEGF inhibitor and chemotherapy only, regardless of PD-L1 expression and EGFR or ALK genetic alteration status (9).

Bispecific antibodies (bsAbs) contain two different antigen-binding sites in one molecule, making it possible to block immune checkpoints and VEGF simultaneously. AK112 is the first anti-PD-1/VEGF bsAbs worldwide. AK112 binds to human VEGF-A, which is involved in tumor angiogenesis, and to human PD-1, a cell surface receptor expressed primarily on activated T cells and inhibits their activation. A phase Ib/II study NCT04900363 demonstrated that AK112 was well-tolerated and presented remarkable anti-tumor efficacy as first or second-line therapy for advanced NSCLC was reported in 2022 ASCO. HB0025 is another bsAbs targets both PD-L1 and VEGF. The *in vitro* and *in vivo* data from preclinical studies indicate HB0025 offers the potential for significant clinical benefits. Both AK112 and HB0025 are continued for further research and testing in clinical trials (10).

Identifying predictive biomarkers to optimize the benefit of angiogenesis inhibitors remains an ongoing challenge (11). This study takes angiogenesis-associated genes (AAGs) as an entry point to investigate the association among angiogenesis, tumor microenvironment, and clinical outcome. We systematically analyzed the expression of 36 AAGs and their effect on lung adenocarcinoma patients’ development, prognosis, and TME. We attempt to elucidate the reason of certain lung adenocarcinoma may not respond to immunotherapy. Our results indicate that *JAG1* expression is a biomarker for a worse prognosis, and patients with *JAG1* expression might benefit from a combination of immunotherapy with angiogenesis inhibitor or bispecific antibodies able to block VEGF.

## Methods

### Data collection and procession

**Figure S1** showed a flowchart of this study. The Lung adenocarcinoma (LUAD) mRNA expression profiles and related clinical data were collected from The Cancer Genome Atlas (TCGA) data portal. The mRNA expression data were transformed to values in transcripts per million (TPM). Gene Expression Omnibus (GEO) databases: GSE135222 and GSE126044 were downloaded from the GEO repository. Angiogenesis-associated genes (AGG) were obtained from GSEA Molecular Signatures Database (MSigDB-hallmark gene sets), a total of 36 genes in “HALLMARK_ANGIOGENESIS” pathway was extracted from the hallmark sub database.

### Identification of angiogenesis-associated group

Unsupervised clustering was used to separate patients into two groups, named angiogenesis-associated subtypes (AGSs)]. Next, a Kaplan-Meier (K-M) plot was developed to measure the OS differences. AGG that met the criterion of false discovery rate (FDR) <0.05 between AGS1 and AGS2 were identified as differentially expressed, and enrichment analysis was performed subsequently.

### Identify the immune landscape of AGS

Infiltrating stromal cells and immune cells constitute the main part of normal cells in tumor tissue. They not only disrupt tumor signals in molecular research, but also play an important role in cancer biology. Based on “Estimating stromal cells and immune cells in tumors using expression data” (ESTIMATE) - a method that uses gene expression characteristics to infer the proportion of stromal cells and immune cells in tumor samples, we calculated the stromal and immune scores of AGSs.

Next, we obtained five immune expression signatures: wound healing (W.H), macrophage regulation (M.R), lymphocyte infiltration (Lym. Inf), IFN-γ response (IFN-γ), and TGF-β response (TGF-β); C1-C6 immune subtypes of each TCGA LUAD samples from Thorsson et al. (12). Moreover, we compared them between AGSs. Considering that immune infiltration was related to measures of DNA damage, we compared several measures of DNA damage, including fraction alteration, number of segments, aneuploidy score (AS), homologous recombination defects (HRD), and intratumor heterogeneity (ITH), which also achieved from Thorsson et al.

### The description of AGSs' mutation landscape

The whole-exome sequencing (WES) data obtained from the TCGA database was used to describe the mutation spectrum and interaction between AGSs, and then searched for specific hypermutation. Next, we calculated the tumor mutation burden (TMB) score with exons uniformly counted as 40M regions and compared it between two AGSs.

### Compare the immune microenvironment of AGSs

We performed ssGSEA to calculate scores of immune cells, whose markers were achieved from TISIDB (http://cis.hku.hk/TISIDB/), and compared their scores between AGSs. To assess the immune state for AGSs, we compared the expression of the following marker genes of myeloid-derived suppressor cells, which have been proven causes the immunosuppressive microenvironment: *CD33*, *ITGAM*, *OLR1*, *S100A9* (13, 14).

### Metabolic capacity assessment and drug susceptibility analysis

We collected four major metabolic pathways from MSigDB and previous studies (15, 16), including glycolysis, fatty acid oxidation, pentose phosphate pathway, and glutamate metabolism (**Table S1**).

Furthermore, we used the “pRRophetic” package (17) to predict the treatment response of each LUAD sample, which was determined by the half maximal inhibitory concentration (IC50) based on the Genomics of Drug Sensitivity in Cancer (GDSC) database.

### Identification and validation of hub genes

The Univariate and multivariate cox regression analysis was performed to delineate the prognostic gene signature. Then, we analyzed the correlation of signature gene expression and measures of immune state, including lymphocyte infiltration and TGF-β pathway activation. Finally, *JAG1* was selected as signature gene because its expression was significantly negatively correlated with lymphocyte infiltration and positively with TGF-β pathway.

To validate the function of *JAG1*, we analyzed the correlation between its expression and markers of basal cells, including endothelial cells: *PECAM1*, *CD34*, *ICAM1*, *PTPRC*; fibroblast cell: *LUM*, *DCN*, *COL1A1*; mesenchymal stem cells: *ZEB1*, *MME*, *ANPEP*, *ITGB1*, *THY1*, *TCF4*, and *SOX2*. Furthermore, we divided the patients of TCGA LUAD datasets into two groups based on *JAG1* expression, samples with expression greater than the mean value were classified as *JAG1*-high group, otherwise as *JAG1*-low group. Then we compared their scores of glycolysis and fatty acid oxidation between *JAG1*-high and *JAG1*-low groups. Finally, we analyzed the *JAG1* expression in two immunotherapy cohorts. We also performed a correlation analysis between *JAG1* and immunotherapy signatures expression including *CD274* (PD-L1) and *CTLA4* (18).

### The description of immune landscape at single-cell transcriptome level

The single-cell transcriptome data obtained from GSE131907, which included 208,506 cells derived from 58 lung adenocarcinomas from 44 patients, which covered primary tumor, lymph node and brain metastases, and pleural effusion in addition to normal lung tissues and lymph nodes. 12 of them were primary tumor samples. In order to reduce the heterogeneity among samples, we selected them for the following analysis. The single-cell transcriptome data were preprocessed using the “Seurat” package, and the cell annotation used results provided by the authors. We calculated percentages of cell types among samples, and analyzed their correlation with *JAG1* positive cell rate. To explore the relationship between T cell status and *JAG1* expression. We used ProjecTIL to parse human scRNA-seq T cell data in the context of murine TIL profiles (19), and described the atlas of T cells. Then, we researched the relationship between *JAG1* positive ratio and subtype percentage. Furthermore, we counted the proportion of each subgroup of T cells in *JAG1* positive and negative cells, and compared the T cell subgroup composition in them. Finally, we calculated the active scores of four metabolism pathways, then compared them between *JAG1* positive and negative cells.

### Cell-cell communication analysis between JAG1-positive and negative samples

First, the positive rate of *JAG1* at the single-cell level was not considerable overall, so we observed the distribution of *JAG1*-positive cells among various cells types by *JAG1* staining. Considering that the clinical assay usually detects on tumor cells, we divided the samples into two groups by the proportion of positive cells in the epithelium. The cell-cell interaction strength between the two groups was analyzed by the “Cellchat” R package, which predicted major signaling inputs and outputs for cells and how those cells and signals coordinate for functions using network analysis and pattern recognition approaches (20). Further, based on the ligand receptor database, significant signal flow and ligand-receptor interaction in the positive group were found out.

### Statistical analyses

All analyses were performed using R software (version 3.6.1). Differences between groups were evaluated using Wilcoxon rank-sum tests for continuous data and Fisher’s exact tests for categorical variables. Pearson’s test was used for the correlation analysis. The center point-based partitioning method with the pam() function in the “cluster” package was used to perform unsupervised clustering. ssGSEA scores were calculated using the GSVA package. K-M plots were constructed using the Kaplan–Meier “survival” package. All analyses were two-sided, and statistical significance was set at *P* < 0.05.

## Results

### Identification of AGS dependent on angiogenesis-associated genes

**Figure 1:**
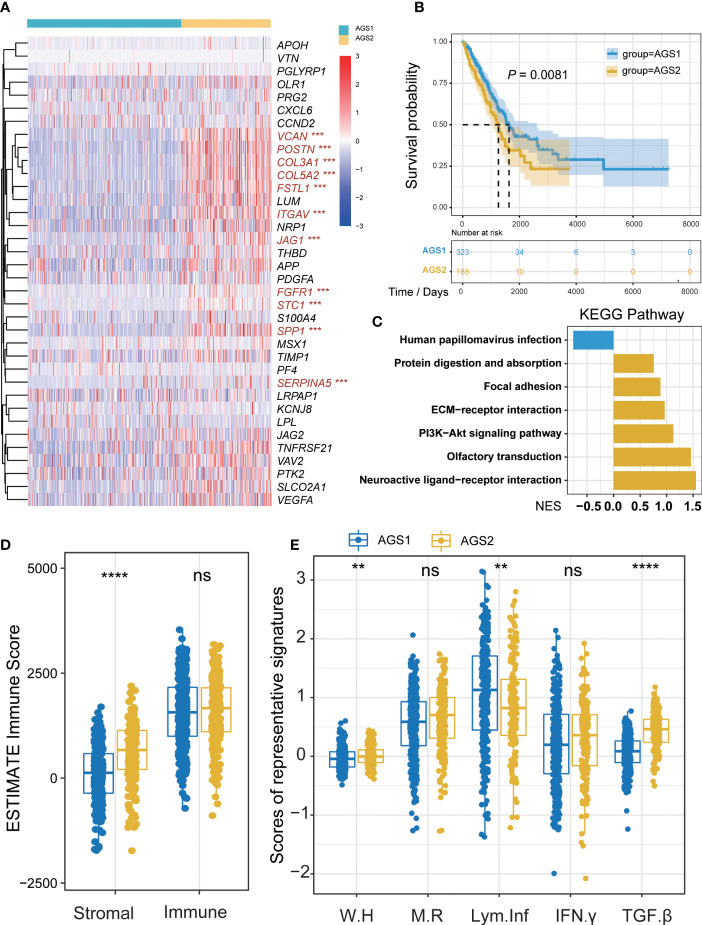
Identification of AGS clusters. **(A)** The heatmap of 26 AGGs expression in AGS1 and AGS2. The significantly different expressed genes were colored by red. **(B)** The Kaplan–Meier plot of the comparison in the overall survival between two clusters. **(C)** The KEGG enrichment pathway of different genes between AGS1 and AGS2. **(D)** The boxplot of Stromal and immune score calculated by ESTIMATE between AGS1 and AGS2. **(E)** The boxplot of five immune expression signatures scores calculated by ssGSEA between AGS1 and AGS2. *, P < 0.05; **, P < 0.01; ***, P < 0.001; ****, P < 0.0001. ns represents p value is not significant.

Firstly, we divided lung adenocarcinoma patients into two groups based on 36 angiogenesis-associated genes, which we named angiogenesis-associated subtypes (AGSs). 11 genes of AGGs showed significant differences between two AGSs, including *JAG1*, *VCAN*, *POSTN*, *COL3A1*, *COL5A2*, *FSTL1*, *ITGAV*, *FGFR1*, *STC1*, *SPP1*, and *SERPINA5* (*P*_all_ < 0.05, **Figure 1A**). Overall, AGS2 exhibited an abundant enrichment of AGGs. Importantly, we found that patients with AGS2 had a poorer prognosis than those with AGS1 (**Figure 1B**), and enrichment analysis showed AGS2 significantly active PI3K-Akt signaling pathway and epithelial mesenchymal transition (**Figure 1C**, **Figure S2A**). Additionally, we compared tumor purity and immune scores between the AGSs, and the results showed that patients in AGS2 had higher stromal scores (*P* < 0.05) but no difference in immune scores (**Figure 1D**). More importantly, we analyzed the five immune expression signatures between AGSs, and found that AGS2 had significantly lower lymphocyte infiltration, accompanied with higher TGF-β response, which indicated AGS1 is an immune “hot” phenotype, while AGS2 is an immune “cold” phenotype (*P*_all_ < 0.05, **Figure 1E**). All above mentioned suggested that angiogenesis-associated genes enrichment may reprogram the “cold” phenotype of tumor immune microenvironment, thus leading to different survival outcomes.

### Prognosis-worse AGS2 exhibited an immunosuppressive state on transcriptome and genomic levels

**Figure 2:**
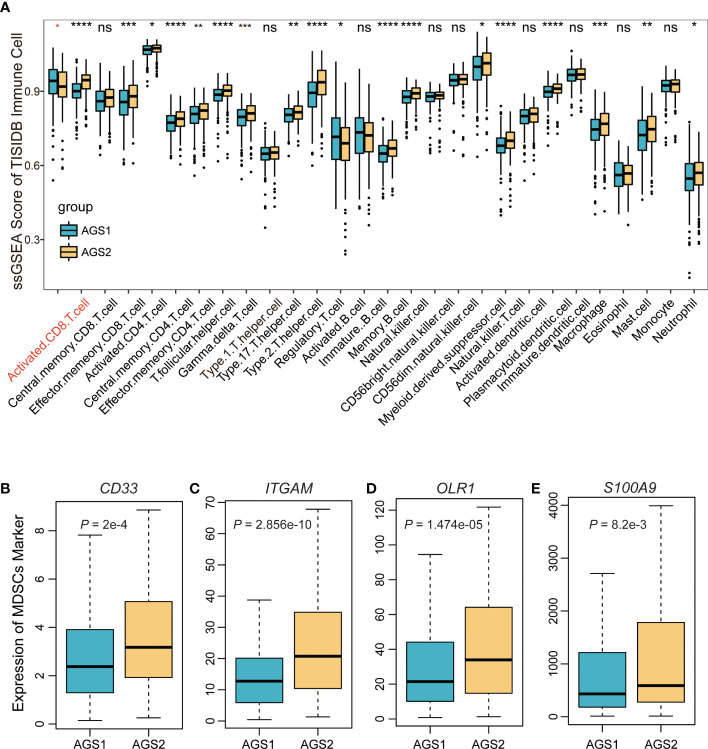
The immune landscape of AGS clusters. **(A)** The boxplot of the ssGSEA score of 29 immune cells based on markers from the TISIDB database. **(B-E)** The boxplot of MDSC markers: **(B)** CD33, **(C)** ITGAM, **(D)** OLR1, **(E)** S100A9. *, P < 0.05; **, P < 0.01; ***, P < 0.001; ****, P < 0.0001. ns represents p value is not significant.

To research the competence of tumor microenvironment, we calculated the ssGSEA score of 28 immune cells based on markers from the TISIDB database. The comparison results showed that AGS2 exhibited obviously lower activated CD8 T cells, and a variety of other immune cells also showed significant differences (**Figure 2A**). Considering the importance of myeloid-derived suppressor cells (MDSCs), which were known to suppress immune responses by inhibiting T cell proliferation and activation, we compared markers of MDSCs between AGSs. We found that *CD33*, *ITGAM*, *OLR1*, and *S100A9* were significantly expressed higher in AGS2 (*P* = 2×10^-4^, 2.86×10^-10^, 1.47×10^-10^, 8.2×10^-3^, respectively, **Figures 2B–E**).

**Figure 3:**
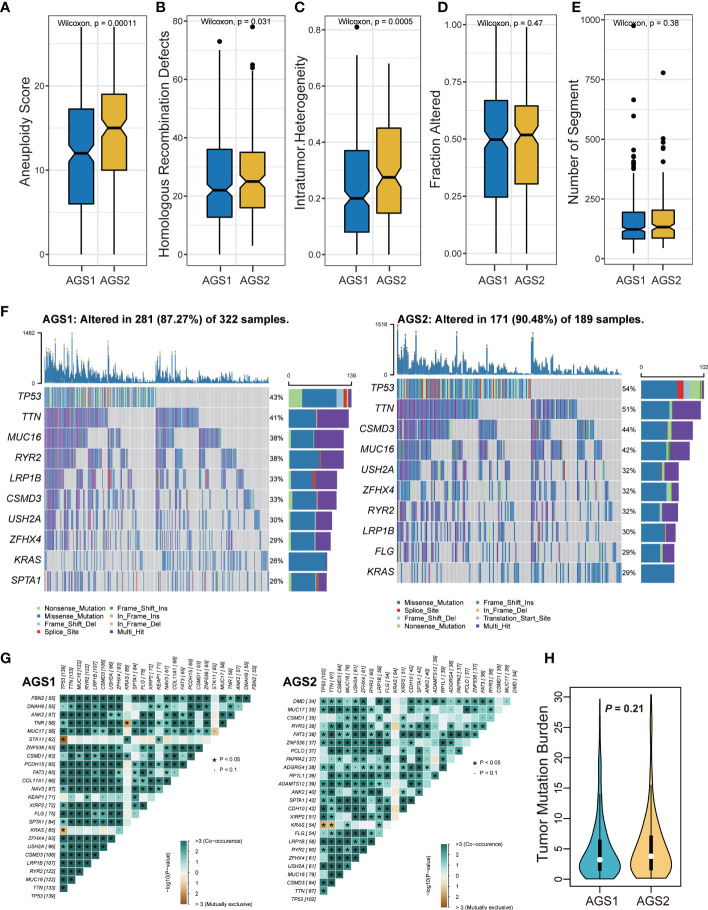
The genomic landscape of AGS clusters. **(A-E)** Measures of DNA damage, **(A)** aneuploidy score, **(B)** homologous recombination defects, **(C)** intratumor heterogeneity, **(D)** Fraction altered, and **(E)** number of segments between AGS1 and AGS2. **(F)** The mutation spectrum of two clusters. **(G)** The mutation correlation of two clusters. **(H)** The violin plot of tumor mutation burden between AGS1 and AGS2.

The comparison of DNA damage measures, including AS, HRD, ITH, altered fraction, and number of segments, were all significantly stronger in AGS2 than in AGS1, representing a poorer prognosis of tumor patients with immunosuppressive state (*P*_all_ < 0.05, **Figures 3A–E**). Next, we described the mutation spectra of AGSs, and revealed several high-frequency mutations. For example, FLG was specifically mutated in AGS2 (**Figure 3F**). Additionally, the mutation interactions were weaker in AGS2 (**Figure 3G**). There was no difference in TMB between AGSs (**Figure 3H**).

### Significant activation of glycolysis may induce immunosuppressive myeloid cells

**Figure 4:**
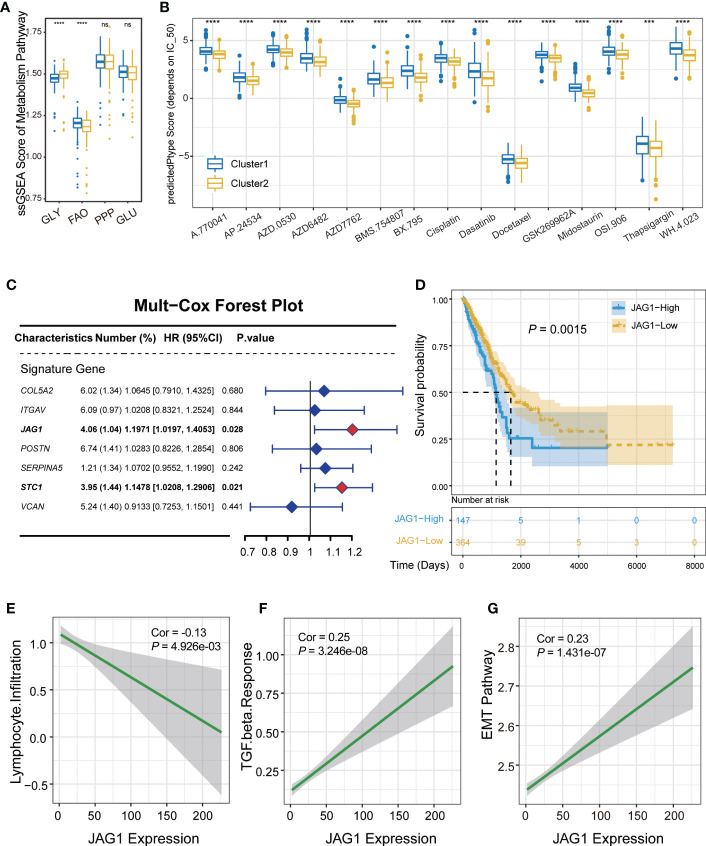
Validation of the correlation between *JAG1* and immune microenvironment. **(A)** The boxplot of metabolism pathway activation between AGS1 and AGS2. **(B)** The boxplot showed IC50 value representing drug responds. **(C)** The forest plot of multivariate Cox regression. **(D)** The Kaplan–Meier plot of the comparison in the overall survival between AGS1 and AGS2. **(E-G)** The scatter plot of *JAG1* expression and **(E)** lymphocyte infiltration, **(F)** TGF-β response, and EMT pathway. *, P < 0.05; **, P < 0.01; ***, P < 0.001; ****, P < 0.0001. ns represents p value is not significant.

Considering that metabolic activation could affect the tumor microenvironment, we compared scores of four major metabolism pathways between AGSs. The result demonstrated that glycolysis is obviously activated in AGS2. Several studies have shown that tumor cells tend to select the metabolic mode of glycolysis, which leads to the accumulation of lactic acid in the microenvironment, thereby promoting the function of immunosuppressive cells such as TAMs, MDSCs and Treg (13). At the same time, fatty acid oxidation was suppressed in AGS2 (*P*_all_ < 0.05, **Figure 4A**). Drug susceptibility prediction was performed using “pRRophetic” package based on the GDSC database. Screening of drugs sensitive to ARG2 group patients, and the result showed that patients in AGS2 were more sensitive to 15 drugs than ARG1, such as such as ABT 263 (navitoclax), cisplatin, dasatinib AP.24534 (ponatinib), and midostaurin (*P*_all_ < 0.05, **Figure 4B**).

### JAG1 expression was negatively correlated with lymphocyte infiltration as a marker gene of AGS2

Multivariate regression analysis was performed to identify hub regulatory factors. *JAG1* and *STC1* showed significant effect on prognosis (hazard ratio (HR): 1.20, *P* = 0.028; HR: 1.15, *P* = 0.021, respectively, **Figure 4C**). Kaplan–Meier plot of the comparison in the overall survival between *JAG1*-high and *JAG1*-low groups shown in **Figure 4D**. *JAG1*, a ligand of the Notch signaling pathway, regulates cell differentiation and proliferation in various cancers. To verify the relationship between *JAG1* expression level and immune microenvironment, we performed the correlation analysis, which showed that the expression of *JAG1* was obviously negative with lymphocyte infiltration, and positive with TGF-β response (R=-0.13, *P* = 5×10^-3^; R=0.25, *P* = 3.246×10^-8^, respectively, **Figures 4E, F**). Furthermore, the EMT pathway was also positively correlated with *JAG1* expression obviously (R=0.23, P=1.431×10^-7^, **Figure 4G**). However, the expression of *STC1* did not show the same consequence (**Figures S3A, B**). Moreover, the *JAG1* expression was a negative correlation with immune cell score, positive correlation with stromal score, although not significant (R = -0.04, *P* = 0.34; R = 0.06, *P* = 0.16, respectively, **Figures S3C, D**). Assessing the level of *JAG1* in multi-omics, we found that *JAG1* can be detected from either copy number amplification or methylation level (**Figures S3E, F**).

### JAG1-high patients had activated glycolysis and were prone to immunotherapy resistance

**Figure 5:**
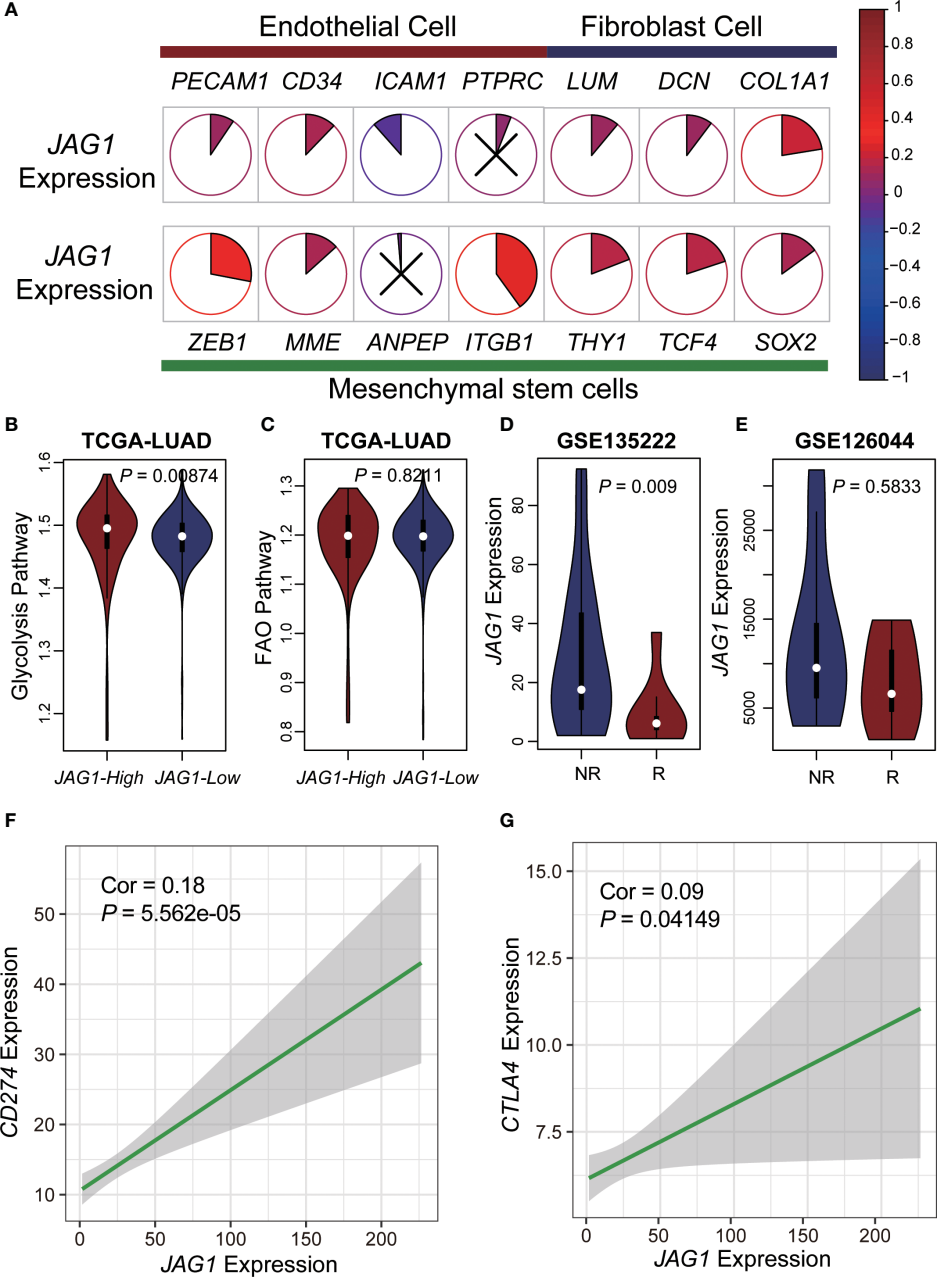
Metabolic characteristics and immunotherapy resistance of *JAG1*-high patients. **(A)** The pie plot showed the correlation of *JAG1* expression and basal cells markers. Colors represented correlation coefficients, blue for negative and red for positive correlations. And, the sector area also represented the correlation. P < 0.05 was considered significant, and those that were not significant were marked with X. **(B, C)** The violin plot of glycolysis and FAO pathway activation. **(D, E)** The expression of *JAG1* between respond and non-respond group in two anti-PD1/PD-L1 immunotherapy cohorts. **(F, G)** The scatter plot of *JAG1*expression and CD274, CTLA4 expression.

Previously, we found more stromal cells in AGS2, and we explored the relationship between markers of various types of stromal cells and *JAG1* expression. The result demonstrated that most markers positively interrelated with *JAG1*, especially mesenchymal stem cells, such as ITGB1 (**Figure 5A**). Next, we compared the activation of glycolysis and FAO pathway between *JAG1*-high and *JAG1*-low groups, which revealed that *JAG1*-high patients had stronger activated glycolysis than *JAG1*-low. (*P* < 0.05, **Figures 5B, C**). In order to explore the efficacy of immunotherapy, we used two datasets of PD1/PD-L1 treatment cohorts to compare the expression of *JAG1* between treatment-sensitive and resistant groups, accompanied with a correlation analysis of *JAG1* expression with PD-L1 and CTLA4 expression. The results indicated that *JAG1*-high patients had higher expression of *PD-L1* and *CTLA4* than *JAG1*-low group (R=0.18, *P* = 5.563×10^-5^; R=0.09, *P* = 4.149×10^-2^, respectively, **Figures 5F, G**). Moreover, *JAG1*-high patients were resistant to pure anti-PD-L1/anti-PD1 therapy (**Figures 5D, E**). The treatment resistance is likely a glycolysis-mediated immunosuppressive microenvironment resulting from angiogenesis.

### Patients with a higher proportion of JAG1 positive cells had less T cell infiltration, and most were Treg cells

**Figure 6:**
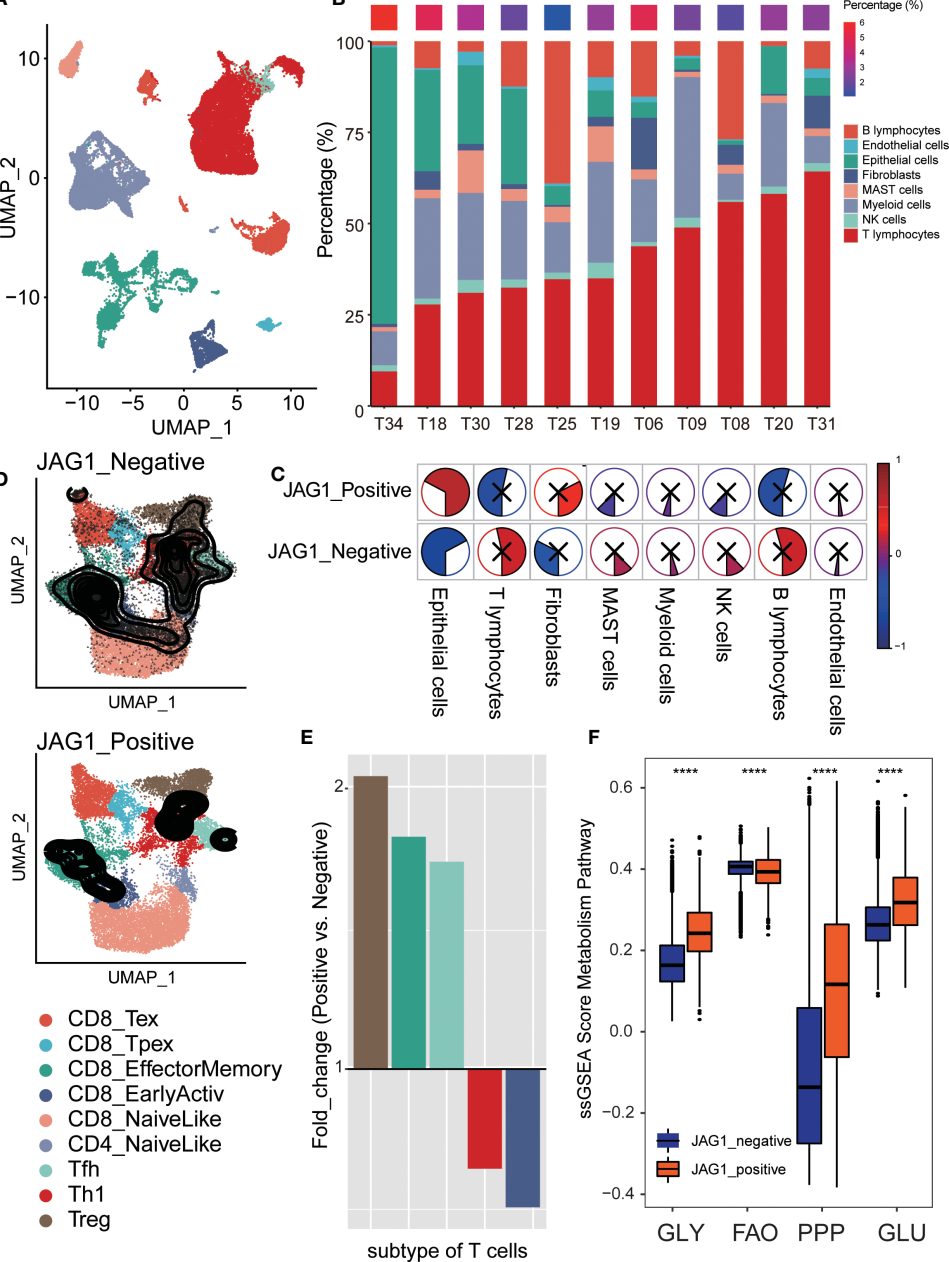
Comparison of Treg proportion between JGA1+ and JAG- cells in single-cell level. **(A)** The TSNE clustering visualization of single-cell RNA sequencing of lung adenocarcinoma colored by cell types. **(B)** The composition ratio of each cell type per sample. The percentage of JAG+ cells was showed above colored from blue (low) to red (high). **(C)** The pie plot showed the correlation of JGA1+ cell proportion and each cell type’s composition ratio. Colors represented correlation coefficients, blue for negative and red for positive correlations. And, the sector area also represented the correlation. P < 0.05 was considered significant, and those that were not significant were marked with X. **(D)** The tSNE plot of T cells colored by subtype and divided into two groups based on *JAG1* expression: *JAG1*+ and *JAG1*- group. **(E)** The fold change of T cell subset composition of positive and negative cells. **(F)** The comparison of metabolism pathway activation between *JAG1*+ and *JAG1*- cells. *, *P* < 0.05; **, *P* < 0.01; ***, *P* < 0.001; ****, *P* < 0.0001.

To explore T cell status at the single-cell level accurately, we described the single-cell transcriptome atlas of lung adenocarcinoma from GSE131907. The cell annotation was downloaded from the original research. Firstly, we counted the compositional proportions of cell types among samples and sorted them by the proportion of T lymphocytes. Explore the relationship between the rate of *JAG1*+ cells and T lymphocytes, we found that the patients with a higher percentage of *JAG1*+ cells were mainly characterized by lower T cell infiltration (**Figures 6A, B**). Comparing the correlation between the rate of *JAG1*+ cells and the proportion of each cell type, we found that it was significantly positively correlated with malignant epithelial cells, and negatively with T lymphocytes, indicating that patients with high *JAG1* expression had higher tumor purity, lower T cell infiltration (**Figure 6C**). Furthermore, we annotated subpopulations of T lymphocytes by ProjecTIL, and divided them into nine subgroups, including CD8+ terminally-exhausted (Tex) effector cluster, CD8+ precursor-exhausted (Tpex) cluster, CD8+ effector memory cluster, CD8+ early active cluster, CD8+ naïve cluster, CD4+ naïve cluster, CD4+ follicular-helper (Tfh) cluster, CD4+ Th1-like cells, and a cluster of regulatory T cells (Treg). The TSNE plot showed that the distribution densities of various T cell types in the *JAG1*+ and JAG- subsets were quite different (**Figure 6D**). At the same time, we calculated the fold change value of the proportion of T cell subsets in *JAG1*+ and *JAG1*- subsets, which manifested that Treg is enriched in the JAG+ subgroup, CD8+ early active cells are enriched in the *JAG1*- subgroup (**Figure 6E**). Finally, we evaluated the activation of metabolic pathways between *JAG1*+ and *JAG1*- groups, and found that glycolysis, pentose phosphate pathway, and glutamate metabolism activated special in *JAG1*+ cells, while fatty acid oxidation was activated in *JAG1*- cells (**Figure 6F**).

### JAG1-positive epithelial cells produced immunosuppressive interactions with various cell types

**Figure 7:**
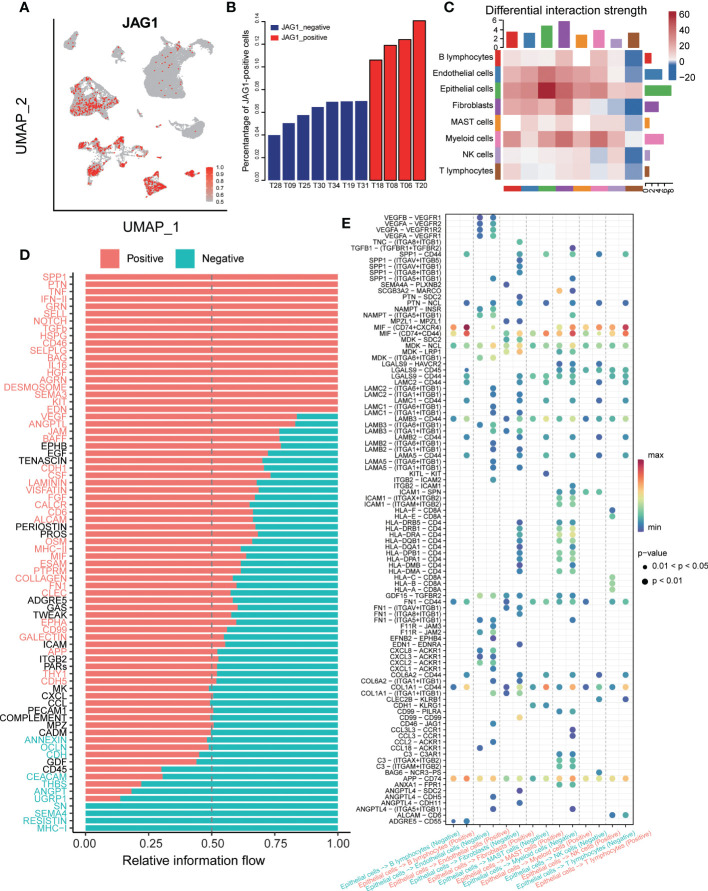
The cell-cell communication between *JAG1* positive and negative samples. **(A)** The TSNE clustering visualization of *JAG1* expression. **(B)** Histogram showed the composition of positive cell among 11 samples, ordered by percentage of *JAG1*+ cells. **(C)** The differential interaction strength of *JAG1*positive and negative samples. Red represents stronger interaction in the *JAG1* positive group, Blue represents stronger interaction in the *JAG1* negative group. **(D)** Significantly activated signal flow between *JAG1* positive and negative samples. **(E)** Dot plot showed significantly different ligand-receptor interaction between two groups.

To dissect the interactions of the tumor microenvironment, we explored the intercellular communication between the JAG-positive and *JAG1*-negative groups. First, we stained the expression of *JAG1* on umap plot, and the results showed that: *JAG1*+ cells were mainly distributed in epithelial cells, endothelial cells, fibroblasts and myeloid cells (**Figure 7A**). Next, we counted the proportion of *JAG*+ cells in epithelial cells in each sample and sorted them. Seven samples had a very low proportion of positive cells (<10%), and we defined them as *JAG1*-negative samples and the rest as *JAG1*-positive samples (**Figure 7B**). Then, we compared the cell-cell interaction strength between the two groups. Red represented the stronger interaction in the *JAG1*-positive samples in the heatmap. The results showed that the *JAG1*-positive samples had stronger cell-cell communication (**Figure 7C**). Furthermore, we classified the ligand-receptor interaction and explored the signal flow that was significantly activated between the two groups. The results showed that most signal flows in the *JAG1* positive samples was stronger, including: SPP1 and SELL signal flow, which related to immunosuppressive microenvironment, and tumor angiogenesis-related pathway: NOTCH, VEGF, FGF signal flows (**Figure 7D**). Finally, we explored the significantly activated ligand-receptor interaction in *JAG1*-positive samples. The result showed that the HLA-A, HLA-B, HLA-C of epithelial cells, was significantly strongly interacted with CD8A of T cells in *JAG1*-negative samples, but not in *JAG1*-positive samples. Moreover, SPP1- (ITGAV+ITGB5), SPP1-(ITGAV+ITGB1), and SPP1-(ITGA8+ITGB1), which was associated with immunosuppressive, occurred in *JAG1*-positive group. The CD46-*JAG1* ligand-receptor interaction occurred in epithelial cells and endothelial cells was obvious in *JAG1*-positive group, which suggested that targeting CD46 in tumor cells may decrease the expression of *JAG1*, thereby reducing the immunosuppressive phenotype (**Figure 7E**).

## Discussion

Tumorigenesis-associated genetic alterations have been classified into eight distinctive and complementary biologic capabilities, one of which is inducing angiogenesis. Tumor microenvironment (TME) widely contributes to tumorigenesis, apparently including pathological angiogenic process (21, 22). At the same time, tumor cells promote angiogenesis and inflammation to evade surveillance and clearance by the immune system (23).

In this study, we explored the prognostic value of AGGs in LUAD. We observed different gene expression levels between the two AGSs groups with significantly different prognostic outcomes, and found that AGGs enriched in the prognosis-worse group: AGS2 and the opposite in the prognosis-favorable AGS1. There were 11 genes expressed differently significantly between the two AGSs. Another pan-cancer study calculated risk scores to evaluate AGGs’ prognostic value, in which unfavorable genes for patient prognosis included all the 11 genes we found (24).

Although immune scores showed no difference between groups but immune expression signatures calculated by ssGSEA showed AGS2 had significantly lower lymphocyte infiltration (dominated by T and B cells) and a higher TGF-βresponse. Absence or exclusion of T cells in the tumor parenchyma character a cold phenotype (25). Nearly every cell in the tumor microenvironment uniquely responds to TGF-β, which plays complex roles in tumorigenesis, including angiogenesis and immunosuppression (26–28).

Herein, we explored transcriptome and genomic levels of AGSs. Prognosis-worse AGS2 exhibited lower activated CD8 T cells, a key determining the probability of clinical response to cancer immunotherapies (29), by comparison of 28 immune cells ssGSEA score between groups. Furthermore, the indicators pointing to immunosuppression showed higher expression in AGS2, including MDSCs-associated markers (*CD33*, *ITGAM*, *OLR1*, *S100A9*) and DNA damage-associated measures (including AS, HRD, ITH).

Metabolic reprogramming refers to a shared set of pathways observed in highly proliferative tumors or cancer cells, which is a hallmark of cancer as it is beneficial to the initiation, proliferation, invasion, and metastasis of tumors in the TME (30). The comparison of four metabolism pathways scores between AGSs showed AGS2 exhibited an obviously activated glycolysis pathway. Aerobic glycolysis (the Warburg effect) (31) has been widely accepted as a common feature of metabolic reprogramming, which is a preference for glycolysis and lactate secretion in the presence of oxygen, promoting the function of immunosuppressive cells (13).

All the findings above indicated and partly verified AGS2 group is an immune “cold” phenotype, which may respond poorly to immune checkpoint blockade (ICB) monoclonal antibodies. Drug susceptibility prediction results showed ARG2 group was more sensitive to 15 drugs, including cisplatin, which is one of the platinum regimens. Although targeted therapy and immunotherapy are all the rage now, platinum regimens still take a crucial place in chemotherapy for incurable NSCLC (32). Therefore, patients with similar characteristics of AGS2 may choose cisplatin among various Platina regimens according to our study.

Several high-frequency mutations were revealed. The prognosis-better AGS2 group had FLG as a specific mutation in our study. FLG mutations had been proved as a tumor-associated mutations in serval cancers, including gastric cancer (33), skin cutaneous melanoma (34), prostate cancer (35), and cervical cancer (36).

Furthermore, we derived *JAG1* and *STC1* from 11 genes with significant differences in expression between two AGSs by multivariate regression analysis. *JAG1* expression showed negative correlation with lymphocyte infiltration, positive correlation with TGF-βresponse and EMT pathway activation significantly, but *STC1* did not. We finally decided *JAG1* was a biomarker gene for ARG2 group, which represented unfavorable prognosis and immunosuppression.

*JAG1* exists as an essential Notch ligand that triggers Notch signaling through cell-cell interactions to promote tumor growth and has been reported to correlate with a poor clinical prognosis in multiple carcinomas, such as hepatocellular carcinoma (37), gastric carcinoma (38), breast carcinoma (39), ovarian carcinoma (40). All the studies indicated a negative correlation between *JAG1* and cancer prognosis. Multi-omics analysis in this study exhibited that *JAG1* can be detected from either copy number amplification or methylation level.

We previously found more stromal cells in the AGS2 group, which are determinants of the TME, nurture tumor cells, contribute to tumor growth and metastasis, and also influence anti-tumor immunity (41). Most markers of stromal cells positively interrelated with *JAG1* expression in our study, especially mesenchymal stem cells. We further explored the correlation between metabolic reprogramming and different *JAG1* expression level, which showed *JAG1*-high expression group had stronger activated glycolysis than *JAG1*-low.

As immunotherapy has become prospering in the treatment of lung cancer and we found that *JAG1* high expression may have an immunosuppressive microenvironment in this study. We further explored *JAG1* as a predictive biomarker for ICB treatment response by comparing the expression level of *JAG1* between treatment-sensitive and resistant groups in two datasets of PD1/PD-L1 treatment cohorts. *JAG1*-high exhibited resistance to anti-PD-L1/anti-PD1 monotherapy, with simultaneously high expression of *PD-L1*, *CTLA-4*. In clinical treatment, patients with high *PD-L1* expression were generally considered to be sensitive to immunotherapy, but our result showed that those patients were resistance. Simultaneous high expression of multiple immune checkpoints was one of the potential causes of resistance. Another important reason may be the immunosuppressive microenvironment caused by glycolysis, according to the previous discussion.

All the findings indicated that targeted *JAG1*/Notch pathway signaling holds potential effective biological therapy for LUAD with *JAG1* high expression. Specific anti-human/rat *JAG1* monoclonal antibodies (mAbs) could inhibit notch signaling *in vitro* in various tumor cell lines, such as 15D11 (42–44), J1-65D and CTX014. Even though none of the mAbs has reported data from clinical trials, they remain promising. AL101 and fosciclopirox were two notch pathway inhibitors that reported preliminary data from phase 1/2 clinical trials in specific carcinomas in the 2022 ASCO annual meeting.

On the other hand, Notch lies downstream of VEGF, and the Notch pathway is involved in a feedback loop with VEGF. They play distinct but complementary roles in tumor angiogenesis (45, 46). In addition, navicixizumab (OMP-305B83) is a bispecific antibody that inhibits the Notch pathway and VEGF pathway; Phase 1a trial data showed preliminary signs of anti-tumor activity in multiple tumor types, particularly in ovarian cancer (47).

At last, we described the immune landscape at the single-cell level accurately by 11 LUAD samples data and got consistent results with those mentioned above, patients with a higher proportion of JAG+ cells had less T cell infiltration, and the most subtype were Treg cells.

We further discovered that *JAG1*+ cells were mainly distributed in epithelial cells, endothelial cells, fibroblasts, and myeloid cells Signal flows associated with the immunosuppressive microenvironment and tumor angiogenesis are stronger in *JAG1*-positive samples, which also had significantly activated ligand-receptor interaction in SPP1-(ITGAV+ITGB5), SPP1-(ITGAV+ITGB1), and SPP1-(ITGA8+ITGB1).

A recent pre-clinical study suggested SPP1 as new potential target could help minimize the immunosuppressive effect of adjuvant chemoradiotherapy. Moreover, a study conducted on mice showed that an increase in the expression of SPP1 in the lung is responsible for the immunosuppressive metastatic niche formation (48, 49). In addition, we observed that *JAG1*-positive samples’ epithelial cells and endothelial cells had more obvious CD46-*JAG1* ligand-receptor interaction. Theoretically feasible to reduce the immunosuppressive phenotype indirectly by targeting CD46 to decrease the expression of *JAG1*.

Our study has several limitations. First, our results were confirmed in both TCGA and GEO databases, it is necessary to validate these results in a multicenter cohort. In addition, we observed data-level correlations among glycolytic activation, the immunosuppressive microenvironment, and poor patient outcomes, but the underlying mechanisms were not elucidated. The regulation of each step involved requires us to conduct more in-depth research in the future. This study merely provides a hypothesis for the immune escape pattern of lung adenocarcinoma patients.

## Conclusion

Our study revealed that angiogenic ability can stratify lung adenocarcinoma patients, and the population with higher *JAG1* expression characterized by poor prognosis, low degree of T lymphocyte infiltration, high TGF-β response, and active metastasis-related EMT pathway. These patients were resistant to anti-PD-1/PD-L1 immunotherapy, the reason may be that angiogenesis leads to the activation of tumor glycolysis, resulting in an immunosuppressive tumor microenvironment. We suggested treatment of anti-angiogenesis therapy with immunotherapy can be considered for these patients to improve prognosis.

## Data availability statement

The original contributions presented in the study are included in the article/**Supplementary Material**. Further inquiries can be directed to the corresponding authors.

## Author contributions

WG, PL, and XT designed the study and reviewed the data. JH, LL, LLL, and XC performed experiments and drafted the manuscript. LL performed bioinformatics analyses. All authors contributed to the article and approved the submitted version.
