# Supplementary material for: JAG1 is correlated to suppressive immune microenvironment and predicts immunotherapy resistance in lung adenocarcinoma
Source: Front Oncol. 2023 Feb 27;13:1091488. doi: 10.3389/fonc.2023.1091488 (PMC10009168; doi:10.3389/fonc.2023.1091488)
Supplement: Supplementary file 1 [file DataSheet_1.docx]

**Angiogenesis-mediated suppressive immune microenvironment leading to poor prognosis and immunotherapy resistance in Lung Adenocarcinoma**

Jing He^1*^, Lu Li^2, 3*^, Xiaoyan Chen^2, 3^, Minghui Ge^2, 3^, Yong Ren^2, 3^, Xinyu Tang^4#^, Ping Liu^1#^, Wen Gao^1#^

^1^Department of Oncology, Jiangsu Province Hospital and Nanjing Medical University First Affiliated Hospital, Nanjing 210029, China.

^2^State Key Laboratory of Translational Medicine and Innovative Drug Development, Jiangsu Simcere Diagnostics Co., Ltd., Nanjing, China.

^3^Nanjing Simcere Medical Laboratory Science Co., Ltd., Nanjing, China.

^4^Department of radiation oncology, Jiangsu Province Hospital and Nanjing Medical University First Affiliated Hospital, Nanjing 210029, China.

**Supplementary Figure legends**

**Figure S1.** The workflow chart of the study.


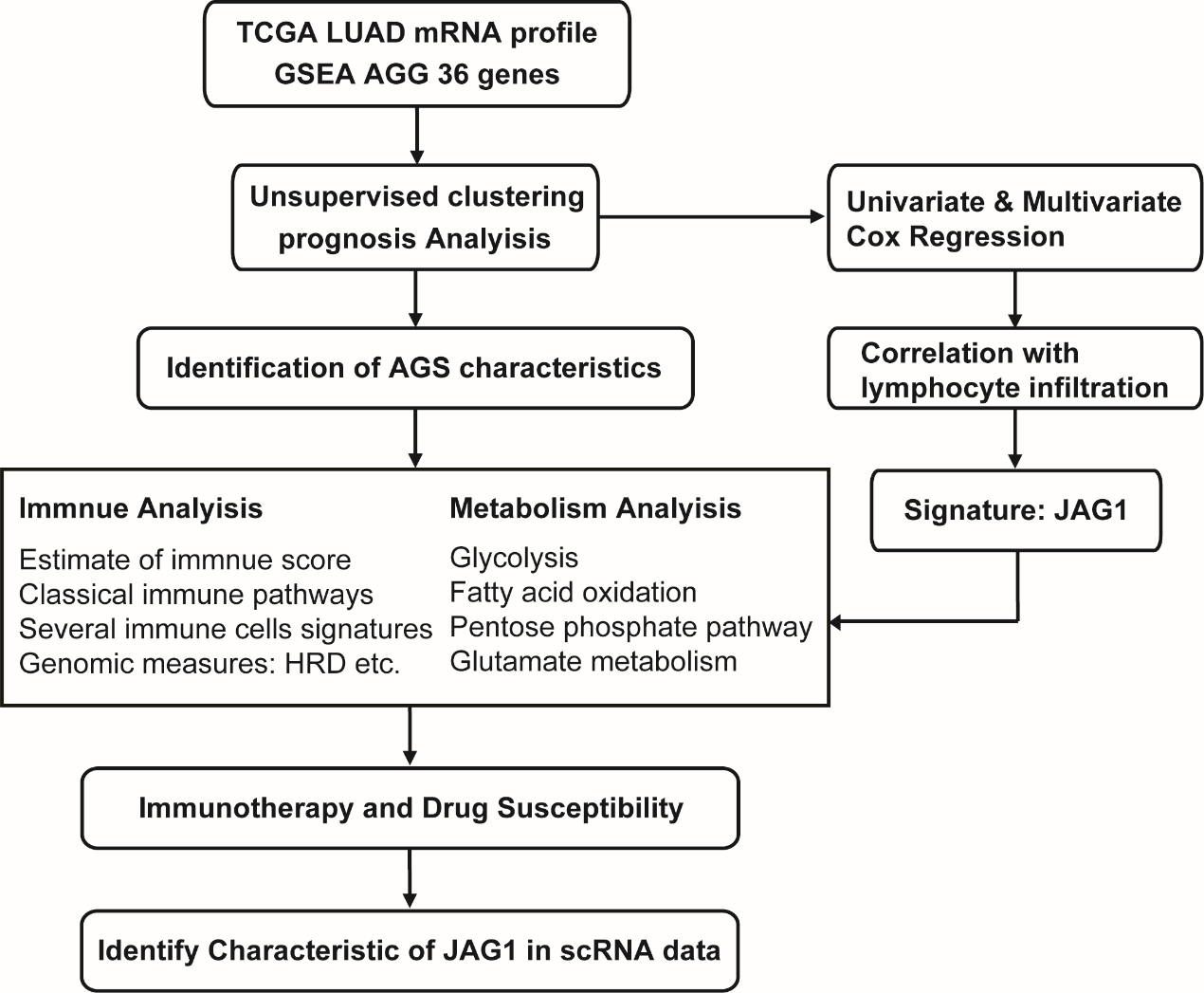


**Figure S2.** The immune landscape of AGS clusters.


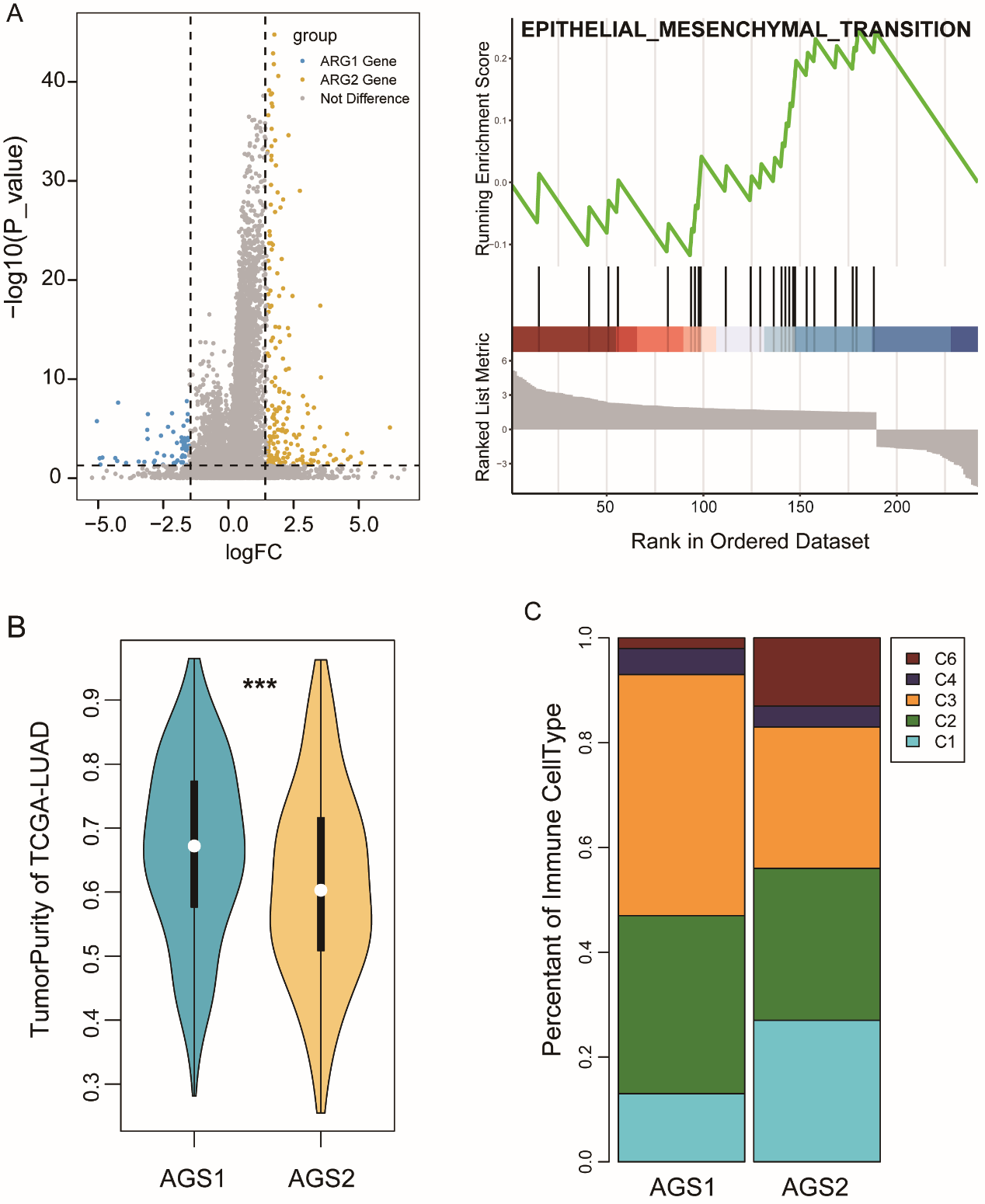


(A) The volcano plot showed significantly differentially expressed genes between the two groups (left). The GSEA enrichment plot of epithelial mesenchymal transition (right). (B) The violin plot of tumor purity in two clusters (C) Proportion of immune subtypes.

**Figure S3.** Characters of the JAG1 in multi-omics levels


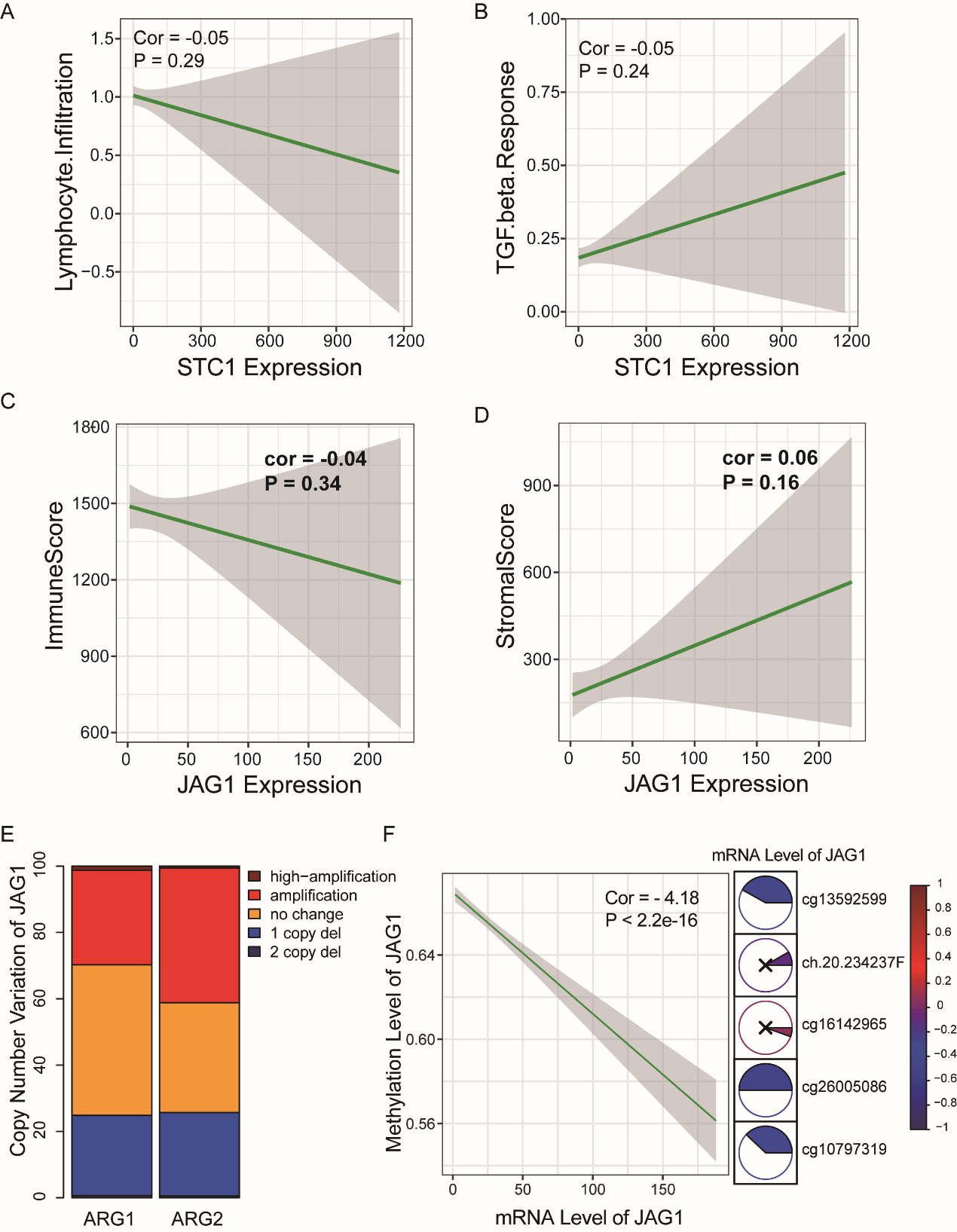


(A-B) The scatter plot of STC1expression and lymphocyte infiltration, TGF-β response. (C-D) The scatter plot of JAG1 expression and immune score, stromal score. (E) The comparison of copy number changes between the two groups. (F) Correlation between transcript level and methylation level of JAG1. The methylation of the five JAG1 probes is shown on the right. *, P < 0.05; **, P < 0.01; ***, P < 0.001; ****, P < 0.0001.

**Table S1.** Markers of four metabolic pathways from MSigDB and previous studies.
